# Tissue engineering of the gastrointestinal tract: the historic path to translation

**DOI:** 10.1186/s13036-022-00289-6

**Published:** 2022-04-04

**Authors:** Claudia A. Collier, Christian Mendiondo, Shreya Raghavan

**Affiliations:** 1 Department of Biomedical Engineering, Texas A&M University, Emerging Technologies Building, 3120 TAMU, College Station, TX 77843 USA; 2 Department of Nanomedicine, Houston Methodist Research Institute, Houston, TX USA

**Keywords:** Tissue engineering, Gastrointestinal tract, Scaffolds, Bioreactors, Organoids, In vitro models, Tissue engineer intestine

## Abstract

The gastrointestinal (GI) tract is imperative for multiple functions including digestion, nutrient absorption, and timely waste disposal. The central feature of the gut is peristalsis, intestinal motility, which facilitates all of its functions. Disruptions in GI motility lead to sub-optimal GI function, resulting in a lower quality of life in many functional GI disorders. Over the last two decades, tissue engineering research directed towards the intestine has progressed rapidly due to advances in cell and stem-cell biology, integrative physiology, bioengineering and biomaterials. Newer biomedical tools (including optical tools, machine learning, and nuanced regenerative engineering approaches) have expanded our understanding of the complex cellular communication within the GI tract that lead to its orchestrated physiological function. Bioengineering therefore can be utilized towards several translational aspects: (i) regenerative medicine to remedy/restore GI physiological function; (ii) in vitro model building to mimic the complex physiology for drug and pharmacology testing; (iii) tool development to continue to unravel multi-cell communication networks to integrate cell and organ-level physiology. Despite the significant strides made historically in GI tissue engineering, fundamental challenges remain including the quest for identifying autologous human cell sources, enhanced scaffolding biomaterials to increase biocompatibility while matching viscoelastic properties of the underlying tissue, and overall biomanufacturing. This review provides historic perspectives for how bioengineering has advanced over time, highlights newer advances in bioengineering strategies, and provides a realistic perspective on the path to translation.

## Introduction

The anatomy and physiology of the gastrointestinal tract (GI) is of poetic complexity, due to its diverse cellular players, integration of neural, immune, secretory, absorptive, and motility signals [1–3]. In addition to the variety of specialized cell types the GI tract contains, its function is also influenced by the presence of the intestinal microbiome, chronic stress, inflammation, and neural regulation via the brain-gut axis [4–6]. The complex nature of the GI tract makes its pathophysiology both challenging to diagnose and treat. Central to intestinal physiology is the mechanics of peristalsis, intestinal motility which occurs from esophagus to anus in varying patterns of propagating contractions and relaxations interrupted by sphincters or valves [7]. Disruptions in peristalsis are central to several GI diseases and disorders, and can even be secondary to diabetes, Parkinson’s disease, and autism spectrum disorders. These disruptions in GI motility often result in a class of disorders known as functional gastrointestinal disorders (FGIDs) since they disrupt the homeostatic functions of the GI tract that include digestion, absorption, luminal transport, and waste disposal [8]. Often, FGIDs manifest as disruptions in quality of life due to symptoms such as abdominal pain, bloating, nausea and vomiting, diarrhea, and constipation. The traditional mainstay to treating GI dysfunction is symptomatic relief with pharmaceuticals, and in severe and specialized clinical cases, surgery. However, in recent times, the advent of stem cell and tissue engineering has brought with it the possibility of repairing intestinal motility and FGIDs with cell-based therapies [9–13]. With the generation of more sophisticated 3D bioengineered models, the field has been able to understand the underlying cell-to-cell interactions and pathophysiology of GI diseases – this will ultimately allow for patients with GI dysfunction to receive targeted, personalized treatments through tissue engineering [14]. It is important to note that FGIDs are but a tiny class of intestinal diseases and disorders, and it is beyond the scope of this mini review to outline the complex pathophysiologies associated with the gut, well-reviewed by several experts [15–20]. Of note, there are also many in depth reviews on advances in bioengineering to create intestinal models to facilitate the study of host-microbial interactions, toxicology, and drug absorption across engineered epithelial layers [21–24]. The focus here is towards restoring intestinal motility/peristalsis, and bioengineering advances towards that.

Over the last 20 years, many animal models have been developed for FGID [25, 26]. Progressively, these models evolved to mimic key features of conceptual FGID models that are triggered by centrally targeted stimuli (neonatal stress, post-traumatic stress disorder) or those triggered by peripherally targeted stimuli (infection, inflammation) [27, 28]. These models have even thus evolved into using transgenic and knockout animals, as well as the demonstration of predictive legitimacy in terms of responsiveness to candidate drugs [28]. While in vivo models replicate these complex interactions, they are famously difficult for variable isolation and specific parameter control for translational and fundamental discoveries [21, 29–31]. Originally described in 1994 [32], the development of tissue-engineered intestine has now been shown in both small and large animal models [33–36]. It is here that bioengineered in vitro models offer a pathway to simplify and break down in vivo variables and study them in well-controlled conditions for observation of cell response or physiology. Advances in tissue engineering has led from 2D monolayers of tissue culture into 3D cultures of self-organized systems that closely mimic in vivo physiology of the gut and the application of bioreactors to incorporate a closer microenvironment of the GI. Of note, the key pathophysiological feature of FGIDs is disruptions in intestinal motility – therefore, this will be the focus of our mini-review.

In this review, we will dive deep into various tissue engineering models relevant to FGIDs with recent advances within the intestinal tract, challenges of tissue engineering (both historic and current), as well as exploring what comes after in vitro modeling and the translation of these results into the clinical studies.

## Key cellular players of gastrointestinal motility

**Fig. 1:**
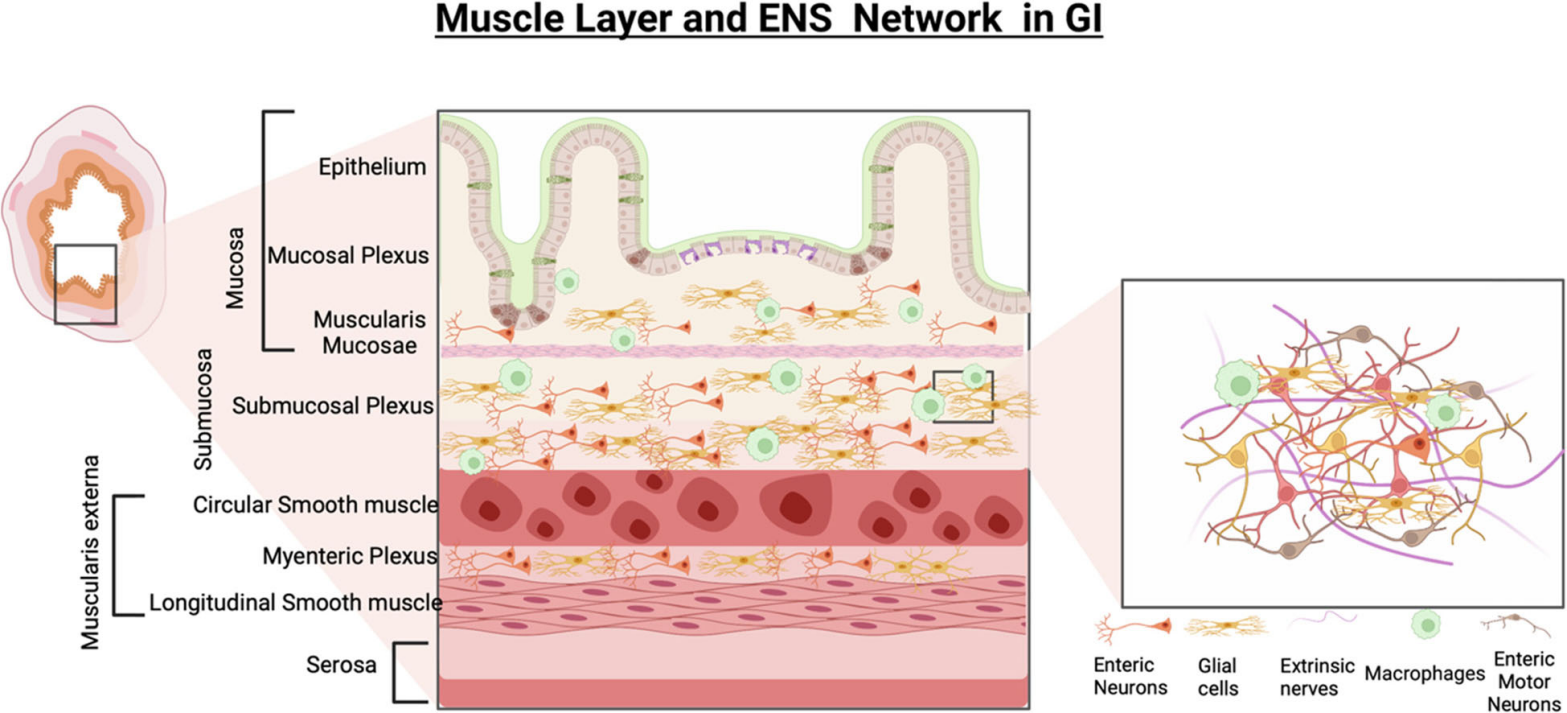
Schematic of the complex system of muscle layers, immune cells, and enteric nervous system within the gastrointestinal tract

The gastrointestinal tract is composed of four general concentric layers (from the innermost to the outermost): the mucosa, submucosa, muscularis externa, and the serosa and contains subcomponent layers that contribute to the structural and functional components [37]. These layers are illustrated in Fig. 1, and broken down into their subcomponents. Located beneath the outermost serosal layer, the muscularis externa layer consists of an inner circular and outer longitudinal muscle layer, which collectively range from 1.1 to 2.4 mm in thickness [37–39]. Smooth muscle cells (SMCs) are elongated contractile cells contained in both the circular and longitudinal muscle layers of the muscularis layer of the GI tract and are the driving force behind peristalsis and motility. SMCs must maintain a mature, contractile phenotype and be properly oriented (i.e., concentrically in circular layers, and elongated axially in longitudinal layers) in order to produce coordinated GI contractions of a physiologically appropriate magnitude [40–42]. Coordination between the outer longitudinal and inner circular SMC layers is important to result in coordinated multiaxial propagating strain (contractile and relaxant portions) of the peristaltic wave. This is the trickiest portion in GI tissue engineering, ensuring the orientation of the two SMC layers relative to one another is of high fidelity, and the maintenance of the contractile phenotype of SMCs.

The enteric nervous system (ENS) regulates contraction and relaxation of the SMCs via an intrinsic neural network that combines excitatory, inhibitory and interneurons [40, 43]. The ENS is organized into two plexuses: the myenteric plexus is sandwiched between the outer longitudinal SMC layer and the inner circular SMC layer; while the submucosal plexus sits between the inner circular SMC layer and the epithelial/mucosal layers of the gut [44, 45]. The myenteric plexus coordinates SMC contraction and the propulsion of intestinal contents, while the submucosal plexus primarily controls intestinal secretion and absorption [44–46]. SMCs and enteric neurons are also in close proximity to interstitial cells of Cajal (ICCs), which act as the “pacemakers of the gut” [47]. Slow waves generated and propagated by ICCs serve the purpose of depolarizing and polarizing the membrane potentials of SMCs via voltage dependent Ca^2+^ channels, adding to the electromechanical coupling [48]. These slow waves add an additional layer of control over the rhythmic contraction and relaxation of SMCs.

In addition to SMCs, enteric neurons, and ICCs, gastrointestinal function and motility is also influenced by the activity of various immune cells such as macrophages, mast cells, T cells, and natural killer cells that integrate signals from the mucosa. Macrophages are present throughout the entire gastrointestinal tract [49]. Intestinal macrophages phagocytose bacteria and pathogens that attempt to cross the epithelium, and they interface with SMCs and enteric neurons via bone morphogenetic protein 2 (BMP2) and bone morphogenetic protein receptor to regulate colonic contractility and peristalsis, respectively [49, 50]. Mast cells (MCs) are also present throughout all layers of the GI tract and help to maintain intestinal homeostasis [51]. MCs release a variety of mediators during degranulation such as tryptase, histamine, serotonin, and cytokines, which regulate GI function by stimulating epithelial cells, intestinal macrophages, and enteric neurons [52]. T cells mediate intestinal homeostasis and inflammation by interacting with lumen microbes and metabolites, communicating with epithelial cells, and protecting against various pathogens [53]. During intestinal inflammation, T cells invade the muscularis layer of the GI tract from the mucosa and release cytokines (IL-4 and IL-13), amplifying the contractile activity of SMCs [54]. Natural killer (NK) cells also modulate intestinal homeostasis and immune response development through their interactions with other GI cells such as macrophages, T cells, and epithelial cells [55]. Additionally, NK cells produce IFNγ, which likely disrupts intestinal motility by suppressing the contractile activity of SMCs [56]. The neuro-immune level of communication is important to integrate signals from the gut microbiome and the intestinal epithelium to modulate communication [57], but is beyond the scope of this review. The focus of this review remains on the effectors of intestinal motility, namely the smooth muscle layers and their regulatory units.

## Current approaches to GI bioengineering

**Fig. 2:**
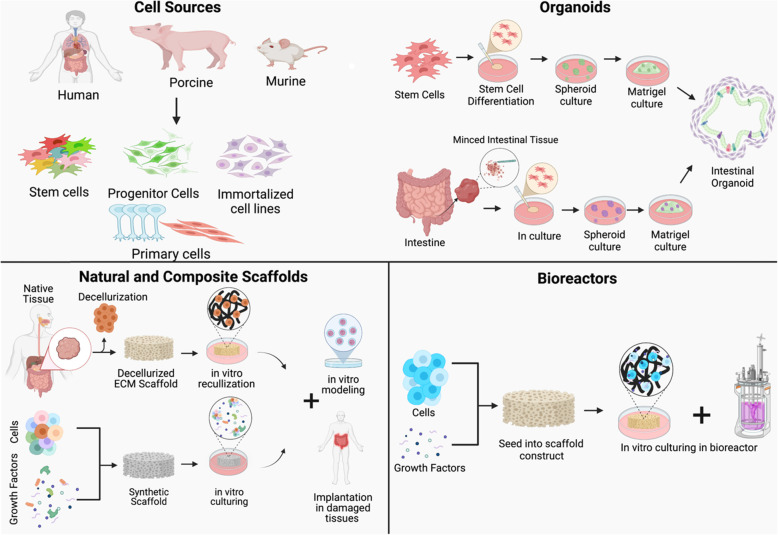
Current GI strategies to engineer tissue engineered intestine include the careful selection of GI cell sources, generation of organoids with natural or composite scaffold support, and bioreactor technologies

Bioengineering within the GI tract can be used for several applications ranging from unraveling cellular interactions that contribute towards physiology or pathophysiology, therapeutic screenings, and regenerative medicine [58]. In the last context, the goal of tissue engineering is to restore, maintain or improve damaged organs or tissue which has recently become increasingly important to develop in tissues that are prone to degeneration, disease, or injury [14, 59]. There are a variety of strategies that can be implemented in tissue engineering, depending on the application or disease modeling such as choosing the correct cell source, modeling organoids with natural or composite scaffolds, and the addition of bioreactor tools illustrated in Fig. 2. Of note, central to the study or restoration of GI function is intestinal motility/peristalsis, which adds a layer of mechanical complexity to the problem [60].

Here, we review the current tools and systems of biomimetic in vitro gastrointestinal models available that have been used to study disease pathophysiology, motility and intestinal physiology, tissue regeneration, cell-cell interaction, cell-matrix interactions.

### Cell sources used within the GI

For in vitro models, the cellular composition is the main determinant of its physiological function and mechanical function to encompass intestinal motility. Choosing cells that can rebuild a 2D layer or 3D layer of the human intestine outside of its physiological environment is the optimal goal. One of the main challenges of utilizing experimental cell biology and tissue engineering in science is the cell source in adult tissues. Cell isolation and expansion of intestinal smooth muscle cells, enteric neuronal and glial cells, ICCs, and/or epithelial cells require complicated enzymatic digestion processes, and significant investment into trophic factors for expansion into adequate numbers and maintenance of cell viability over long culture durations in vitro [23].

#### Primary and immortalized cell lines

Immortalized cell lines have historically been used for in vitro modeling of intestinal epithelium- examples include several human colorectal cancer-derived cell lines like Caco-2 cells [61, 62], HT-29, HT29-MTX, HRA-19 [63]. While the intestinal epithelium is no doubt an important contributor to physiological homeostasis and motility, it is not the effector of motility, which are smooth muscle cells, as described in Section 2. Further, the primary regulator of motility, the enteric neuronal component, has seen the use of immortalized fetal enteric neuronal cells of murine origin in bioengineering. Immortomouse fetal enteric neuronal cells (IM-FENS) have been established from H-2Kb-tsA58 transgenic mice with successful neuronal characteristics similar to primary enteric neurons and have been shown to improve colonic motility [64, 65]. Fetal and adult murine primary enteric neuron cultures have also been established [66–69]. A discussion of stem cell sources for generating a functional ENS is outlined in following sections.

To date, however, there are no immortalized smooth muscle cell lines of human origin available, and therefore, regenerative medicine and GI bioengineering of the muscle layers have relied on other cell sources, including primary and patient-derived sources. Primary cells are isolated directly from the tissue and processed for cell culture and can closely resemble in vivo phenotype [70]. Intestinal stem cells and intestinal organoids are major sources for regenerating the intestinal epithelium and can be sourced from human intestines in abundance. Research has shown that human intestinal enteroids (organoids specific to the intestine) can self-renew, expand indefinitely into intestinal epithelium cell types including enterocytes, goblet cells, Paneth cells and enteroendocrine cells [71]. However, again, while the epithelium is important in recreating the intestinal structure, it plays a limited role in directly affecting intestinal motility.

In many FGIDs, colonic motility has been compromised attributing to symptoms that can impede daily life [8]. As outlined in Section 2, the key cellular effectors of intestinal motility are smooth muscle cells (SMCs), enteric neurons and ICCs. SMCs can be isolated from intestines (murine, rabbit, non-human primate, and human sources) through enzymatic digestions and propagated in culture to attain adequate numbers necessary for 3D bioengineering [40, 72–74]. Early work by Raghavan et al. and Gilmont et al. [75, 76] demonstrated the isolation and bioengineering of SMCs from human intestinal biopsies. Not only did these cultures demonstrate myogenic contractility (i.e., response to exogenously added neurotransmitters), but SMCs also maintained the contractile phenotype, evident by Smoothelin expression. Smooth Muscle cells are the primary cell types within the muscular layers of the bowel wall that facilitate motility [77] and have been implemented into constructs to study peristalsis [78] and motility [23]. In conjunction with SMCs, Interstitial cells of cajal (ICCs) were co-cultured providing intrinsic pacemaker activity [40] and regulation of smooth muscle function and showed to maintain maturity and functionality of both SMCs and ICCs [79]. ICCs are also important cell sources to mediate rhythmic contraction of smooth muscle independently from the ENS with studies displaying the phasic contractions within intestinal organoid tissues, suggesting the phasic contractions are due to ICCs [80].

#### Stem cell-based sources for the GI tract

Mesenchymal stem cells (MSCs) have been another source of cells for GI bioengineering and can be derived from bone marrow [81, 82]. MSCs can differentiate into epithelial cells [83] and smooth muscle cells [84] and act as a cell source for tissue engineering of the small intestine. Current advancements have extended into biomanufacturing MSCs for major expansion in therapeutic use under good manufacturing practices that utilizes assays to ensure the efficacy and quality safety of MSCs [85–88]. Induced pluripotent stem cells (IPSCs) have been utilized to derive enteric neural crest cells resulting in proliferative migratory neuronal and glial cells of damaged intestine tissue [89]. Other sources to target ENS include embryonic stem cells [90, 91], which can differentiate into neural crest cells, neural precursors, and neurons [92–95]. CNS stem cells; experiments using neural stem cells (NSCs) derived from the CNS have strongly supported the idea that stem cells, when transplanted into the intestine, are capable of improving motility disorders [96]. Similar findings were reported in studies using transplantation of neuroepithelial stem cells, derived from the rodent neural tube, into ganglionic rat colon, with the result that gut motility was improved [97].

##### Neural stem cells

Embryonic stem-derived neural precursors have been shown to generate successfully both central and peripheral neurons, glia, enteric neurons, and other neural crest derivatives [98–100]. Another major cell population that aids in intestinal motility are enteric nerve stem cells (ENSCs) which regulate relaxation and contraction of the intestinal wall. ENSCs within the submucosal plexus micromanages the lumen environment and regulates gastrointestinal blood flow as well as controlling the epithelial cell functions and secretion [101]. ENSCs isolated from the intestine have been shown to differentiate into neural crest-derived cell types and ENSCs from the ganglionic colon are able to differentiate into neurons and glia in vivo and ex vivo of chick embryos [102] with intestinal motility assessed in a similar study measuring changes of intraluminal pressure through electric field stimulation (EFS) [97]. A combination of smooth muscle cells and neural stem cells provides the route for developing a functional innervated muscularis layer. Neural crest cells (NCCs), a potential source of autologous neuronal cells, neuroepithelial stem cells, ENS progenitor cells derived from fetal and post-natal mouse and human guts have all been reported to aid in intestinal motility [96, 97, 103–106]. NCCs have also gained popularity in their ability to differentiate into neurons and glial cells with expressed phenotypic markers characterized of the enteric nervous system [107]. Neural crest stem cells (NCSCs) derived from MSCs and human induced pluripotent stem cells (hIPSCs) have been shown to also differentiate into smooth muscle with confirmed phenotypic expression of a-smooth muscle actin and functional enteric-like neurons expressing functional enteric neuron markers [107].

#### The use of organoids in bioengineering

Organoids are defined as an in vitro 3D culture obtained from primary tissue, embryonic stem cells (ESCs) or IPSCs, that are capable of self-organization, multicell-complex and manifest similar organ functionality as the tissue of origin [108, 109]. Organoids have been able to provide the ability to establish a well-controlled system via external manipulation and mimic vivo physiology, respectively. Human intestinal organoids (HIOs) were combined with human-PSC-derived neural crest cells (NCCs) and in vitro results showed migration of NCCs into the mesenchyme, differentiated into neurons and glial cells showing neuronal activity. In vivo engraftment in mice for 6–10 weeks showed formed neuroglial structures similar to a myenteric and submucosal plexus and had functional ICCs [80]. Smooth muscle cells co-cultured with adipose-derived stem cells exhibited improved cell proliferation, contractility, and organoid formation similar to smooth muscle cells [110], rendering human organoids a reliable source of cells to recreate human intestinal structures.

Human pluripotent stem cells have been used to engineer human colonic organoids transplanted into mice where they matured into tissue similar to human colon through RNA sequencing [111] and human gastric organoids which encompassed both the corpus and the antrum [112]. Organoids were implemented to study IBS through cultured 3D embryonic stem cells differentiated into HIOs and in contrast to other organoids, these mature organoids grew with a higher count of mesenchymal cells and used these organoids to test the anti-fibrotic drug, spironolactone, in vitro. Results indicated the clinical and research potential of intestinal organoids as a future model of fibrosis, a model which has thus far been limited to less biologically relevant models and animal models [113]. Although there are a variety of organoids being developed, most of these organoid models only represent single or partial components of a tissue. Bioactive factors or oxygen delivery may not be uniform throughout the system resulting in lacking or undesired differentiation.

### Scaffold-based bioengineering approaches in GI

Scaffolds are architectural support systems in bioengineering, designed to house cells and provide structural integrity of tissue engineered constructs. Scaffolds have been engineered to promote a diverse array of functions that mimic in vivo environments such as structural support, mechanical stability, interactive bioactive cues, reservoir for exogenously applied growth factors and void volume for vascularization or tissue remodeling [114]. In their utility in intestinal tissue engineering with a focus on motility, we review two overarching types of scaffolds: 1) naturally derived/ECM based scaffolds 2) Polymeric and composite scaffolds.

#### Naturally derived/ECM based scaffolds

The ECM is in a state of dynamic reciprocity with resident cells; that is, ECM provides signaling and biophysical cues that influence cell morphology and phenotype [115, 116]. In turn, cells modify their secreted ECM products in response to microenvironmental signals including mechanical stimuli, oxygen, and nutrient concentration [117]. Small intestinal submucosa (SIS) was an early biomaterial scaffold used in GI bioengineering, derived from porcine small intestine incorporates many essential components such as growth factors, glycoproteins, collagen, and proteoglycans [118–120]. Preliminary work tested the implantation of cell-free SIS scaffolds in white rabbit models for the potential of intestinal regeneration, and results showed that by 4 weeks of implantation, the lumen of the SIS regenerated to the small intestinal mucosa including signs of goblet cells and villus like structures [121]. SIS scaffolds also were used in repair of a partial defect created by resection of a portion of the small bowel in canine models which resulted in regeneration of smooth muscle tissue, and the serous membrane with no evidence of intestinal dysfunction or stenosis [122]. A clinical study done on patients suffering with enterocutaneous fistula (ECF), a condition where the intestinal tract develops an abnormal connection with the skin and stomach causing leakage through the skin, underwent a treatment of autologous platelet-rich fibrin glue (PRFG) showing an increase in the time of fistula closure rates [123, 124]. An injectable enzyme-resistant dietary fiber, xanthan, hydrogel was engineered to aid in ECF closure, in contrast to fibrin sealant which also contains anti-digestive properties, the hydrogel offers the opportunity for intestinal gut-barrier function repair and displayed reversible shrinking-swelling characteristics which could aid in the extraction of the hydrogel in vivo [125]. Collagen has also been utilized as a naturally derived scaffold and been shown to successfully show a defined epithelial layer and smooth muscle layer seeded with smooth muscle cells and iPSC derived-smooth muscle progenitor cells (SMPs) [126, 127] and cyclic mechanical testing of collagen scaffold contained more elastin and seeded SMCs exhibited a contractile phenotype [128]. Further study was done on collagen scaffolds seeded with SMCs to investigate the regeneration of endocrine cells and the nerve system in vivo of canine model within the small intestine [129]. Another naturally derived element used in scaffolds is chitosan which have been tested as a support system for bioengineered circular muscle constructs seeded with colonic smooth muscle cells and enteric neural progenitor cells. Differentiated functional neurons were shown through real time force generation and staining showed innervation of SMCs [75, 130–132]. Finally, a 3D bi-layered silk protein scaffold has been utilized as a support ECM for intestinal smooth muscle cells and have been shown to support cell function, differentiation, and neurite growth [133].

With most naturally derived ECM scaffolds, they are fantastic in showing biocompatible and biodegradable characteristics, but they are lacking in control of specific factors that can include batch to batch variability, mechanical strength, and porosity. However, synthetic scaffolds have been widely used as well for their increased mechanical strength, reproducible/controllable mechanical-chemical properties, and controllable biodegradation rates [134] which will be discussed in the next section.

#### Polymeric and composite scaffolds

Polyglycolic acid (PGA) scaffold has been a standard tool of tissue-engineering experiments and has evolved to be commercially available in the form of biodegradable sutures [135–138]. PGA scaffolds have been widely used and offer a unique ability to be optimized for further compatible characteristics of native tissue. For example, tubular PGA scaffolds were prepared by a coating of poly-L-lactic acid (PLLA), then seeded with crypt stem cell organoids and implanted into peritoneal cavity of nude rats. After 4 weeks of implantation, mechanical and porosity properties were assessed of the tissue engineered intestine. Results showed that PLLA coating increased suture retention strength, the peak strength when the surgical wire is pulled out the wall of the tube, and decreased porosity size [139]. PGA scaffolds have also been successfully shown to grow a smooth muscle layer with ability to perform under cyclic strain promoting elastin production and a contractile phenotype similar to native tissue [128, 140–143]. Degradable poly (ethylene glycol) (PEG) hydrogel scaffolds have been utilized to study autologous smooth muscle cell motility phenotype within the synthetic biomaterial. Results showed increased expression of α-smooth muscle actin and myosin including the proliferation of the SMCs on the scaffold [144]. Mesenchymal stem cells were seeded onto a hybrid scaffold consisting of oxidized polyvinyl alcohol (OxPVA) hydrogel cross-linked with decellularized intestinal wall for in vitro study of support cell adhesion and proliferation and in vivo study transplanted in the omentum of rats for 4 weeks for a potential application platform in malabsorption diseases, a disorder where the small intestine cannot absorb enough of certain nutrients and fluids [145]. In 2019, work was considered to improve the mechanical properties of the SIS scaffold by reinforcing the tubular SIS with polylactic-co-glycolic acid (PLGA) nanofibers. This facilitated in giving directional growth support for smooth muscle cells which can be used for a model to study peristalsis [146].

Although scaffolds have proven to be an essential part of studying cellular mechanisms and a steppingstone for mimicking microenvironments, there are limitations that hamper the study of biological activity with the associated microenvironment. Several in vivo studies have focused on large animal models using collagen sponge scaffolds seeded with intestinal smooth muscle to repair patches of intestinal tissue. Despite limited success with formation of epithelial layers, these studies have found it difficult to replicate the alignment and contractile function of smooth muscle cells in vivo which is vital for sufficient nutrient absorption and motility [23, 147, 148]. However, these studies have successfully been able to grow SMCs with expression of α-smooth muscle actin.

### Bioreactor systems

Bioreactors have facilitated cell-based therapies to help maintain well-controlled microenvironments that regulate cell growth, differentiation, and tissue development [80, 149–151]. Through their ability to increase the mass transport of oxygen and nutrients and the role of providing controlled environments for reproducible mechanical forces, including magnitude, frequency, continuous or intermittent, duty cycle, to 3D constructs. Bioreactors have become essential for providing standardized cell-based products or establishing physiologically relevant in vitro models to test pharmacologic agents resulting in different designs of bioreactors with the end goal of cell expansions [152–156]. Even more, bioreactors have an amazing opportunity to be used to study pathophysiological effects of physical forces on developing tissues, and to predict the responses of an engineered tissue to physiological forces upon implantation. In conjunction with biomechanical characterization, bioreactors could help in defining when engineered tissues have a sufficient mechanical integrity and biological responsiveness to be implanted [157]. Simultaneously, gut-on-a-chip systems, evolved from tissue engineering, have been able to establish a minimally functional unit that can recapitulate certain aspects of human physiology in a controlled and straightforward manner [158].

Significant efforts have been made to capture a dynamic microenvironment that compares that of intestinal conditions. The perfusion bioreactor was developed with intestinal organoid units cultured on biodegradable tubular polymer scaffolds with confirmed live cell attachment for 2 days which has some implications for long-term culture and bioengineering of intestinal cells [159]. A more recent bioreactor model attempted to mechanically mimic the contraction and relaxation cycles of the intestine tissue using electro-responsive elastomeric membrane for in vitro modeling [160]. This design better imitated the cyclic peristalsis within the intestine through sinusoidal voltages mimicking amplitude and frequency of intestinal contractions. Pulsatile perfusion bioreactors have been utilized to increase SMC and collagen production seeded onto 3D PLCL scaffolds subjected to pulsatile strain and shear stress for up to 8 weeks. Smooth muscle phenotype expression was measured through *α*-smooth muscle actin and found to be upregulated by a 2.5-fold compared to smooth muscle cultured in static conditions [161]. Another study focused on intestinal smooth muscle periodic contraction facilitated through spontaneous Ca^2+^ oscillation. The constructs were examined with expression of ICC, SMC, and neuronal markers, and more than 3-fold cell growth. Periodic contraction directionality of period constriction, and frequency of rhythmic contractions [79]. Bioreactors have also been utilized to differentiate adipose derived stem cells (an autologous cell source) into smooth muscle cells of decellularized scaffolds increasing the SMC phenotype expression and studying the contraction phenotypes upon collagen gel plating [162].

## Future perspectives and the path to translation

The path to clinical translation of bioengineered intestinal constructs is certainly not as tenuous as it seemed even a decade ago. Some efforts have progressed into the clinical trials, where earlier animal studies confirmed the therapeutic efficacy of bone marrow derived MSC in treating colitis [163, 164], and clinical trials in humans also validated their safety and some positive effect in Crohn’s disease [165–167]. Simultaneously, many SIS constructs have been broadly accepted and in several clinical applications [168–174]; however, many other SIS constructs, including most tissue engineering models discussed within this review, are still in the in vitro and pre-clinical phases for intestinal disease application of regenerative medicine within intestinal motility disorders. Despite the success of tissue engineered strategies in preclinical translational research, very few have had success in the clinical marketplace which can be a result of unmet clinical needs [175, 176]. Efficacy and efficiency of new bioengineered solutions must be carefully evaluated before clinical trials, with careful consideration of clinical benchmarking during pre-clinical evaluation [177, 178]. One of the main challenges of utilizing experimental cell biology and tissue engineering in science is the cell source and cell expansion of intestinal cells require numerous isolations to expand to adequate numbers, and maintain cell viability and phenotypic stability for longer durations [23]. In this context, standardization and quality control of biomanufacturing is a much needed next step that can make bioengineering a common place reality in the clinic. Medical and ethical considerations require intense preclinical investigations of new biomedical products before introduction into clinical applications. Similarly, tissue engineering also requires comparable testing and development strategies including the establishment of rigorous regulatory standards for bioengineered product quality and physiological functionality. With expansion of clinical benchmarking standards for performance of a bioengineered construct similar to biomaterial devices, expansion and standardization of biomanufacturing, the last ingredient to this recipe is collaboration. A mandatory close collaboration between clinicians and scientists (bioengineers, materials engineers, biologists) is needed to translate basic scientific discoveries in tissue engineered gut therapies to shorten the path of bioengineering in the intestine to the clinic.

## Data Availability

Since no data was generated as a part of this manuscript, all data referenced here is publicly available through the PubMed database.

## Abbreviations

ECF: Enterocutaneous fistula
ECM: Extracellular matrix
ENS: Enteric nervous system
ENSC: Enteric neural stem cells
FGID: Functional Gastrointestinal Disorders
GI: Gastrointestinal
HIO: Human intestinal organoids
HIPSC: Human induced pluripotent stem cells
MC: Mast Cells
MSC: Mesenchymal stem cells
NCSCs: Neural crest-derived stem cells
NK: Natural Killer cells
PGA: Poly glycolic acid
PLLA: Poly-l-lactic acid
SIS: Small intestinal submucosa
SMC: Smooth muscle cell

## References

[1] Beverly Greenwood-van Meerveld. Spring, Gastrointestinal Pharmacology; 2017.

[2] Cryan JF, O'Riordan KJ, Cowan CSM, Sandhu KV, Bastiaanssen TFS, Boehme M (2019). The microbiota-gut-brain Axis. Physiol Rev.

[3] Tait C, Sayuk GS (2021). The brain-gut-Microbiotal Axis: a framework for understanding functional GI illness and their therapeutic interventions. Eur J Intern Med.

[4] Jiang Y, Greenwood-Van Meerveld B, Johnson AC, Travagli RA (2019). Role of estrogen and stress on the brain-gut axis. Am J Physiol Gastrointest Liver Physiol.

[5] Molina-Torres G, Rodriguez-Arrastia M, Roman P, Sanchez-Labraca N, Cardona D (2019). Stress and the gut microbiota-brain axis. Behav Pharmacol.

[6] Khlevner J, Park Y, Margolis KG (2018). Brain-gut Axis: clinical implications. Gastroenterol Clin N Am.

[7] Otterson MF, Sarr MG (1993). Normal physiology of small intestinal motility. Surg Clin North Am.

[8] Corazziari E (2004). Definition and epidemiology of functional gastrointestinal disorders. Best Pract Res Clin Gastroenterol.

[9] Burra P, Bizzaro D, Ciccocioppo R, Marra F, Piscaglia AC, Porretti L (2011). Therapeutic application of stem cells in gastroenterology: an up-date. World J Gastroenterol.

[10] Piscaglia AC, Novi M, Campanale M, Gasbarrini A (2008). Stem cell-based therapy in gastroenterology and hepatology. Minim Invasive Ther Allied Technol.

[11] Hotta R, Natarajan D, Burns AJ, Thapar N (2011). Stem cells for GI motility disorders. Curr Opin Pharmacol.

[12] Stamp LA (2017). Cell therapy for GI motility disorders: comparison of cell sources and proposed steps for treating Hirschsprung disease. Am J Physiol Gastrointest Liver Physiol.

[13] Young HM (2005). Neural stem cell therapy and gastrointestinal biology. Gastroenterology.

[14] Zakhem E, Raghavan S, Suhar RA, Bitar KN (2019). Bioengineering and regeneration of gastrointestinal tissue: where are we now and what comes next?. Expert Opin Biol Ther.

[15] Mearin F, Malfertheiner P (2017). Functional Gastrointestinal Disorders: Complex Treatments for Complex Pathophysiological Mechanisms. Digest Dis (Basel, Switzerland).

[16] Holtmann G, Shah A, Morrison M (2017). Pathophysiology of functional gastrointestinal disorders: a holistic overview. Dig Dis.

[17] Drossman DA (2005). Functional GI disorders: what’s in a name. Gastroenterology.

[18] Christensen J (1992). Pathophysiology of the irritable bowel syndrome. Lancet.

[19] Drossman DA (2016). Functional Gastrointestinal Disorders: History, Pathophysiology, Clinical Features, and Rome IV. Gastroenterology.

[20] Fukudo S, Kuwano H, Miwa H (2012). Management and pathophysiology of functional gastrointestinal disorders. Digestion.

[21] Costa J, Ahluwalia A (2019). Advances and Current Challenges in Intestinal in vitro Model Engineering: A Digest. Front Bioeng Biotechnol.

[22] Rocha FG, Whang EE (2004). Intestinal tissue engineering: from regenerative medicine to model systems. J Surg Res.

[23] Bitar KN, Raghavan S (2012). Intestinal tissue engineering: current concepts and future vision of regenerative medicine in the gut. Neurogastroenterol Motil.

[24] Dosh RH, Jordan-Mahy N, Sammon C, Le Maitre CL (2018). Tissue engineering laboratory models of the small intestine. Tissue Eng B Rev.

[25] Accarie A, Vanuytsel T (2020). Animal Models for Functional Gastrointestinal Disorders. Front Psychiatr.

[26] Camilleri M, Buéno L, Andresen V, De Ponti F, Choi M-G, Lembo A (2016). Pharmacologic, Pharmacokinetic, and Pharmacogenomic Aspects of Functional Gastrointestinal Disorders. Gastroenterology.

[27] Mayer EA, Collins SM (2002). Evolving pathophysiologic models of functional gastrointestinal disorders. Gastroenterology.

[28] Haier J, Schmidt F. Fundamentals of Tissue Engineering and Regenerative Medicine. Berlin: Springer; 2009. p. 773–9.

[29] Shanks N, Greek R, Greek J (2009). Are animal models predictive for humans?. Philos Ethics Humanit Med.

[30] Lorian V (1988). Differences between in vitro and in vivo studies. Antimicrob Agents Chemother.

[31] Godbey WT, Atala A (2002). In vitro systems for tissue engineering. Ann N Y Acad Sci.

[32] Vacanti JP, Morse MA, Saltzman WM, Domb AJ, Perez-Atayde A, Langer R (1988). Selective cell transplantation using bioabsorbable artificial polymers as matrices. J Pediatr Surg.

[33] Grikscheit TC, Siddique A, Ochoa ER, Srinivasan A, Alsberg E, Hodin RA (2004). Tissue-engineered small intestine improves recovery after massive small bowel resection. Ann Surg.

[34] Sala FG, Kunisaki SM, Ochoa ER, Vacanti J, Grikscheit TC (2009). Tissue-engineered small intestine and stomach form from autologous tissue in a preclinical large animal model. J Surg Res.

[35] Barthel ER, Speer AL, Levin DE, Sala FG, Hou X, Torashima Y (2012). Tissue engineering of the intestine in a murine model. J Vis Exp.

[36] Spurrier RG, Grikscheit TC (2013). Tissue engineering the small intestine. Clin Gastroenterol Hepatol.

[37] Patel KS, Thavamani A (2021). Physiology, Peristalsis. StatPearls.

[38] Rao JN, Wang JY (2010). Integrated Systems Physiology: from Molecule to Function to Disease. Regulation of Gastrointestinal Mucosal Growth.

[39] Brasseur JG, Nicosia MA, Pal A, Miller LS (2007). Function of longitudinal vs circular muscle fibers in esophageal peristalsis, deduced with mathematical modeling. World J Gastroenterol.

[40] Sanders KM, Koh SD, Ro S, Ward SM (2012). Regulation of gastrointestinal motility--insights from smooth muscle biology. Nat Rev Gastroenterol Hepatol.

[41] Xiu XL, Zheng LF, Liu XY, Fan YY, Zhu JX (2020). Gastric smooth muscle cells manifest an abnormal phenotype in Parkinson's disease rats with gastric dysmotility. Cell Tissue Res.

[42] Huycke TR, Miller BM, Gill HK, Nerurkar NL, Sprinzak D, Mahadevan L (2019). Genetic and Mechanical Regulation of Intestinal Smooth Muscle Development. Cell.

[43] Spencer NJ, Hu H (2020). Enteric nervous system: sensory transduction, neural circuits and gastrointestinal motility. Nat Rev Gastroenterol Hepatol.

[44] Furness JB (2008). The enteric nervous system: normal functions and enteric neuropathies. Neurogastroenterol Motil.

[45] Furness JB (2012). The enteric nervous system and neurogastroenterology. Nat Rev Gastroenterol Hepatol.

[46] Wood JD, Alpers DH, Andrews PLR (1999). Fundamentals of neurogastroenterology. Gut.

[47] Foong D, Zhou J, Zarrouk A, Ho V, O’Connor MD (2020). Understanding the biology of human interstitial cells of Cajal in gastrointestinal motility. Int J Mol Sci.

[48] Sanders KM, Ward SM, Koh SD (2014). Interstitial cells: regulators of smooth muscle function. Physiol Rev.

[49] Viola MF, Boeckxstaens G (2020). Intestinal resident macrophages: multitaskers of the gut. Neurogastroenterol Motil.

[50] Grainger JR, Konkel JE, Zangerle-Murray T, Shaw TN (2017). Macrophages in gastrointestinal homeostasis and inflammation. Pflugers Arch.

[51] Bhuiyan P, Chen Y, Karim M, Dong H, Qian Y (2021). Bidirectional communication between mast cells and the gut-brain axis in neurodegenerative diseases: avenues for therapeutic intervention. Brain Res Bull.

[52] De Winter BY, van den Wijngaard RM, de Jonge WJ (2012). Intestinal mast cells in gut inflammation and motility disturbances. Biochim Biophys Acta.

[53] Ma H, Tao W, Zhu S (2019). T lymphocytes in the intestinal mucosa: defense and tolerance. Cell Mol Immunol.

[54] Akiho H, Lovato P, Deng Y, Ceponis PJM, Blennerhassett P, Collins SM (2005). Interleukin-4- and −13-induced hypercontractility of human intestinal muscle cells-implication for motility changes in Crohn's disease. Am J Physiol Gastrointest Liver Physiol.

[55] Poggi A, Benelli R, Venè R, Costa D, Ferrari N, Tosetti F (2019). Human Gut-Associated Natural Killer Cells in Health and Disease. Front Immunol.

[56] Ford CL, Wang Y, Morgan K, Boktor M, Jordan P, Castor TP (2019). Interferon-gamma depresses human intestinal smooth muscle cell contractility: relevance to inflammatory gut motility disturbances. Life Sci.

[57] Reardon C, Murray K, Lomax AE (2018). Neuroimmune communication in health and disease. Physiol Rev.

[58] Cencic A, Langerholc T (2010). Functional cell models of the gut and their applications in food microbiology--a review. Int J Food Microbiol.

[59] Clevers H, Conder RK, Li VSW, Lutolf MP, Vallier L, Chan S (2019). Tissue-engineering the intestine: the trials before the trials. Cell Stem Cell.

[60] Keller J, Bassotti G, Clarke J, Dinning P, Fox M, Grover M (2018). Advances in the diagnosis and classification of gastric and intestinal motility disorders. Nat Rev Gastroenterol Hepatol.

[61] Hidalgo IJ, Raub TJ, Borchardt RT (1989). Characterization of the human colon carcinoma cell line (Caco-2) as a model system for intestinal epithelial permeability. Gastroenterology.

[62] Hilgers AR, Conradi RA, Burton PS (1990). Caco-2 cell monolayers as a model for drug transport across the intestinal mucosa. Pharm Res.

[63] Simon-Assmann P, Turck N, Sidhoum-Jenny M, Gradwohl G, Kedinger M (2007). In vitro models of intestinal epithelial cell differentiation. Cell Biol Toxicol.

[64] Anitha M, Joseph I, Ding X, Torre ER, Sawchuk MA, Mwangi S (2008). Characterization of fetal and postnatal enteric neuronal cell lines with improvement in intestinal neural function. Gastroenterology.

[65] Raghavan S, Gilmont RR, Miyasaka EA, Somara S, Srinivasan S, Teitelbaum DH (2011). Successful implantation of bioengineered, intrinsically innervated, human internal anal sphincter. Gastroenterology.

[66] Holland-Cunz S, Bainczyk S, Hagl C, Wink E, Wedel T, Back W (2004). Three-dimensional co-culture model of enterocytes and primary enteric neuronal tissue. Pediatr Surg Int.

[67] Zhang Y, Hu W, Amini S, White MK (2013). Mouse enteric neuronal cell culture. Neuronal cell culture: methods and protocols.

[68] Brun P, Akbarali HI, Skaper SD (2018). Culture of neurons and smooth muscle cells from the myenteric plexus of adult mice. Neurotrophic factors: methods and protocols.

[69] Metzger M, Bareiss PM, Danker T, Wagner S, Hennenlotter J, Guenther E (2009). Expansion and Differentiation of Neural Progenitors Derived From the Human Adult Enteric Nervous System. Gastroenterology.

[70] Jensen C, Teng Y (2020). Is It Time to Start Transitioning From 2D to 3D Cell Culture?. Front Mol Biosci.

[71] Chen Y, Zhou W, Roh T, Estes MK, Kaplan DL (2017). In vitro enteroid-derived three-dimensional tissue model of human small intestinal epithelium with innate immune responses. PLoS One.

[72] Tokita Y, Akiho H, Nakamura K, Ihara E, Yamamoto M (2015). Contraction of gut smooth muscle cells assessed by fluorescence imaging. J Pharmacol Sci.

[73] Batista Lobo S, Denyer M, Britland S, Javid FA (2007). Development of an intestinal cell culture model to obtain smooth muscle cells and myenteric neurones. J Anat.

[74] Carnicelli V, Di Giulio A, Romano G, Bozzi A, Oratore A, Delle Fave G (2000). Regional differences in signalling transduction pathways among smooth muscle cells from rabbit colon. Cell Signal.

[75] Zakhem E, Raghavan S, Gilmont RR, Bitar KN (2012). Chitosan-based scaffolds for the support of smooth muscle constructs in intestinal tissue engineering. Biomaterials.

[76] Gilmont RR, Somara S, Srinivasan S, Raghavan S, Bitar KN (2009). S1797 Co-Culture of Enteric Neuronal Cells with Bioengineered Three-Dimensional Colonic Circular Smooth Muscle Constructs. Gastroenterology.

[77] Krezalek MA, Alverdy JC (2016). The role of the microbiota in surgical recovery. Curr Opin Clin Nutr Metab Care.

[78] Bitar KN, Zakhem E (2013). Tissue engineering and regenerative medicine as applied to the gastrointestinal tract. Curr Opin Biotechnol.

[79] Kobayashi M, Khalil HA, Lei NY, Wang Q, Wang K, Wu BM (2018). Bioengineering functional smooth muscle with spontaneous rhythmic contraction in vitro. Sci Rep.

[80] Workman MJ, Mahe MM, Trisno S, Poling HM, Watson CL, Sundaram N (2017). Engineered human pluripotent-stem-cell-derived intestinal tissues with a functional enteric nervous system. Nat Med.

[81] Pittenger MF, Mackay AM, Beck SC, Jaiswal RK, Douglas R, Mosca JD (1999). Multilineage potential of adult human mesenchymal stem cells. Science.

[82] Ferrari G, Cusella-De Angelis G, Coletta M, Paolucci E, Stornaiuolo A, Cossu G (1998). Muscle regeneration by bone marrow-derived myogenic progenitors. Science.

[83] Choi RS, Vacanti JP (1997). Preliminary studies of tissue-engineered intestine using isolated epithelial organoid units on tubular synthetic biodegradable scaffolds. Transplant Proc.

[84] Gu W, Hong X, Le Bras A, Nowak WN, Issa Bhaloo S, Deng J (2018). Smooth muscle cells differentiated from mesenchymal stem cells are regulated by microRNAs and suitable for vascular tissue grafts. J Biol Chem.

[85] Mizukami A, Swiech K (2018). Mesenchymal Stromal Cells: From Discovery to Manufacturing and Commercialization. Stem Cells Int.

[86] Aijaz A, Li M, Smith D, Khong D, LeBlon C, Fenton OS (2018). Biomanufacturing for clinically advanced cell therapies. Nat Biomed Eng.

[87] Roh KH, Nerem RM, Roy K (2016). Biomanufacturing of therapeutic cells: state of the art, current challenges, and future perspectives. Annu Rev Chem Biomol Eng.

[88] Ma T, Tsai A-C, Liu Y (2016). Biomanufacturing of human mesenchymal stem cells in cell therapy: influence of microenvironment on scalable expansion in bioreactors. Biochem Eng J.

[89] Chang DF, Zuber SM, Gilliam EA, Nucho L-MA, Levin G, Wang F (2020). Induced pluripotent stem cell-derived enteric neural crest cells repopulate human aganglionic tissue-engineered intestine to form key components of the enteric nervous system. J Tissue Eng.

[90] Burns AJ, Thapar N (2014). Neural stem cell therapies for enteric nervous system disorders. Nat Rev Gastroenterol Hepatol.

[91] Finkbeiner SR, Freeman JJ, Wieck MM, El-Nachef W, Altheim CH, Tsai Y-H (2015). Generation of tissue-engineered small intestine using embryonic stem cell-derived human intestinal organoids. Biol Open.

[92] Liu Q, Spusta SC, Mi R, Lassiter RN, Stark MR, Höke A (2012). Human neural crest stem cells derived from human ESCs and induced pluripotent stem cells: induction, maintenance, and differentiation into functional schwann cells. Stem Cells Transl Med.

[93] Zhang SC, Wernig M, Duncan ID, Brüstle O, Thomson JA (2001). In vitro differentiation of transplantable neural precursors from human embryonic stem cells. Nat Biotechnol.

[94] Wichterle H, Lieberam I, Porter JA, Jessell TM (2002). Directed differentiation of embryonic stem cells into motor neurons. Cell.

[95] Dhara SK, Stice SL (2008). Neural differentiation of human embryonic stem cells. J Cell Biochem.

[96] Schäfer KH, Micci MA, Pasricha PJ (2009). Neural stem cell transplantation in the enteric nervous system: roadmaps and roadblocks. Neurogastroenterol Motil.

[97] Liu W, Wu RD, Dong YL, Gao YM (2007). Neuroepithelial stem cells differentiate into neuronal phenotypes and improve intestinal motility recovery after transplantation in the aganglionic colon of the rat. Neurogastroenterol Motil.

[98] Pomp O, Brokhman I, Ben-Dor I, Reubinoff B, Goldstein RS (2005). Generation of peripheral sensory and sympathetic neurons and neural crest cells from human embryonic stem cells. Stem Cells.

[99] Bibel M, Richter J, Lacroix E, Barde YA (2007). Generation of a defined and uniform population of CNS progenitors and neurons from mouse embryonic stem cells. Nat Protoc.

[100] Achilleos A, Trainor PA (2012). Neural crest stem cells: discovery, properties and potential for therapy. Cell Res.

[101] Nezami BG, Srinivasan S (2010). Enteric nervous system in the small intestine: pathophysiology and clinical implications. Curr Gastroenterol Rep.

[102] Cheng LS, Hotta R, Graham HK, Belkind-Gerson J, Nagy N, Goldstein AM (2017). Postnatal human enteric neuronal progenitors can migrate, differentiate, and proliferate in embryonic and postnatal aganglionic gut environments. Pediatr Res.

[103] Martucciello G, Brizzolara A, Favre A, Lombardi L, Bocciardi R, Sanguineti M (2007). Neural crest neuroblasts can colonise aganglionic and ganglionic gut in vivo. Eur J Pediatr Surg.

[104] Almond S, Lindley RM, Kenny SE, Connell MG, Edgar DH (2007). Characterisation and transplantation of enteric nervous system progenitor cells. Gut.

[105] Bondurand N, Natarajan D, Thapar N, Atkins C, Pachnis V (2003). Neuron and glia generating progenitors of the mammalian enteric nervous system isolated from foetal and postnatal gut cultures. Development.

[106] Lindley RM, Hawcutt DB, Connell MG, Almond SL, Vannucchi MG, Faussone-Pellegrini MS (2008). Human and mouse enteric nervous system neurosphere transplants regulate the function of aganglionic embryonic distal colon. Gastroenterology.

[107] Li W, Huang L, Zeng J, Lin W, Li K, Sun J (2018). Characterization and transplantation of enteric neural crest cells from human induced pluripotent stem cells. Mol Psychiatry.

[108] Creff J, Malaquin L, Besson A (2021). In vitro models of intestinal epithelium: toward bioengineered systems. J Tissue Eng.

[109] Fatehullah A, Tan SH, Barker N (2016). Organoids as an in vitro model of human development and disease. Nat Cell Biol.

[110] Smolar J, Horst M, Salemi S, Eberli D (2020). Predifferentiated smooth muscle-like adipose-derived stem cells for bladder engineering. Tissue Eng Part A.

[111] Múnera JO, Sundaram N, Rankin SA, Hill D, Watson C, Mahe M (2017). Differentiation of Human Pluripotent Stem Cells into Colonic Organoids via Transient Activation of BMP Signaling. Cell Stem Cell.

[112] Broda TR, McCracken KW, Wells JM (2019). Generation of human antral and fundic gastric organoids from pluripotent stem cells. Nat Protoc.

[113] Rodansky ES, Johnson LA, Huang S, Spence JR, Higgins PD (2015). Intestinal organoids: a model of intestinal fibrosis for evaluating anti-fibrotic drugs. Exp Mol Pathol.

[114] Chan BP, Leong KW (2008). Scaffolding in tissue engineering: general approaches and tissue-specific considerations. Eur Spine J.

[115] Schultz GS, Davidson JM, Kirsner RS, Bornstein P, Herman IM (2011). Dynamic reciprocity in the wound microenvironment. Wound Repair Regen.

[116] Bissell MJ, Hall HG, Parry G (1982). How does the extracellular matrix direct gene expression?. J Theor Biol.

[117] Nelson CM, Bissell MJ (2006). Of extracellular matrix, scaffolds, and signaling: tissue architecture regulates development, homeostasis, and cancer. Annu Rev Cell Dev Biol.

[118] Badylak SF, Kropp B, McPherson T, Liang H, Snyder PW (1998). Small intestinal submucosa: a rapidly resorbed bioscaffold for augmentation cystoplasty in a dog model. Tissue Eng.

[119] Kropp BP, Rippy MK, Badylak SF, Adams MC, Keating MA, Rink RC (1996). Regenerative urinary bladder augmentation using small intestinal submucosa: urodynamic and histopathologic assessment in long-term canine bladder augmentations. J Urol.

[120] Kropp BP, Eppley BL, Prevel CD, Rippy MK, Harruff RC, Badylak SF (1995). Experimental assessment of small intestinal submucosa as a bladder wall substitute. Urology.

[121] Demirbilek S, Kanmaz T, Özardalı İ, Edalı MN, Yücesan S (2003). Using porcine small intestinal submucosa in intestinal regeneration. Pediatr Surg Int.

[122] Chen MK, Badylak SF (2001). Small bowel tissue engineering using small intestinal submucosa as a scaffold. J Surg Res.

[123] Wu X, Ren J, Gu G, Wang G, Han G, Zhou B (2014). Autologous platelet rich fibrin glue for sealing of low-output enterocutaneous fistulas: an observational cohort study. Surgery.

[124] Wu X, Ren J, Wang G, Wang J, Wang F, Fan Y (2015). Evaluating the use of fibrin glue for sealing low-output enterocutaneous fistulas: study protocol for a randomized controlled trial. Trials.

[125] Huang J, Li Z, Hu Q, Chen G, Ren Y, Wu X (2018). Bioinspired Anti-digestive Hydrogels Selected by a Simulated Gut Microfluidic Chip for Closing Gastrointestinal Fistula. iScience.

[126] Mulorz J, Shayan M, Hu C, Alcazar C, Chan AH, Briggs M (2021). Peri-adventitial delivery of smooth muscle cells in porous collagen scaffolds for treatment of experimental abdominal aortic aneurysm. Biomat Sci.

[127] Nakase Y, Hagiwara A, Nakamura T, Kin S, Nakashima S, Yoshikawa T (2006). Tissue engineering of small intestinal tissue using collagen sponge scaffolds seeded with smooth muscle cells. Tissue Eng.

[128] Kim B-S, Mooney DJ (2000). Scaffolds for engineering smooth muscle under cyclic mechanical strain conditions. J Biomech Eng.

[129] Nakase Y, Nakamura T, Kin S, Nakashima S, Yoshikawa T, Kuriu Y (2007). Endocrine cell and nerve regeneration in autologous in situ tissue-engineered small intestine. J Surg Res.

[130] Zakhem E, Raghavan S, Bitar KN (2014). Neo-innervation of a bioengineered intestinal smooth muscle construct around chitosan scaffold. Biomaterials.

[131] Zakhem E, Bitar KN (2015). Development of chitosan scaffolds with enhanced mechanical properties for intestinal tissue engineering applications. J Funct biomat.

[132] Zakhem E, Elbahrawy M, Orlando G, Bitar KN (2015). Successful implantation of an engineered tubular neuromuscular tissue composed of human cells and chitosan scaffold. Surgery.

[133] Chen Y, Guo C, Manousiouthakis E, Wang X, Cairns DM, Roh TT (2020). Bi-layered Tubular Microfiber Scaffolds as Functional Templates for Engineering Human Intestinal Smooth Muscle Tissue. Adv Funct Mater.

[134] Gervaso F, Sannino A, Peretti GM (2014). The biomaterialist's task: scaffold biomaterials and fabrication technologies. Joints.

[135] Dumitriu S. Polymeric biomaterials, Revised and Expanded. CRC Press; 2001.

[136] Scott G, Gilead D (2012). Degradable polymers: principles and applications.

[137] Pillai CK, Sharma CP (2010). Review paper: absorbable polymeric surgical sutures: chemistry, production, properties, biodegradability, and performance. J Biomater Appl.

[138] Middleton JC, Tipton AJ (2000). Synthetic biodegradable polymers as orthopedic devices. Biomaterials.

[139] Liu Y, Nelson T, Chakroff J, Cromeens B, Johnson J, Lannutti J (2019). Comparison of polyglycolic acid, polycaprolactone, and collagen as scaffolds for the production of tissue engineered intestine. J Biomed Mater Res B Appl Biomater.

[140] Lawrence BJ, Maase EL, Lin H-K, Madihally SV (2009). Multilayer composite scaffolds with mechanical properties similar to small intestinal submucosa. J Biomed Mater Res A.

[141] Eberli D, Filho LF, Atala A, Yoo JJ (2009). Composite scaffolds for the engineering of hollow organs and tissues. Methods.

[142] Kim B-S, Nikolovski J, Bonadio J, Mooney DJ (1999). Cyclic mechanical strain regulates the development of engineered smooth muscle tissue. Nat Biotechnol.

[143] Higgins SP, Solan AK, Niklason LE (2003). Effects of polyglycolic acid on porcine smooth muscle cell growth and differentiation. J Biomed Mater Res Part A.

[144] Adelöw C, Segura T, Hubbell JA, Frey P (2008). The effect of enzymatically degradable poly(ethylene glycol) hydrogels on smooth muscle cell phenotype. Biomaterials.

[145] Grandi F, Stocco E, Barbon S, Rambaldo A, Contran M, Fascetti Leon F (2018). Composite scaffolds based on intestinal extracellular matrices and oxidized polyvinyl alcohol: a preliminary study for a new regenerative approach in short bowel syndrome. Biomed Res Int.

[146] Syed O, Kim JH, Keskin-Erdogan Z, Day RM, El-Fiqi A, Kim HW (2019). SIS/aligned fibre scaffold designed to meet layered oesophageal tissue complexity and properties. Acta Biomater.

[147] Nakase Y, Hagiwara A, Nakamura T, Kin S, Nakashima S, Yoshikawa T, Fukuda KI, Kuriu Y, Miyagawa K, Sakakura C, Otsuji E, Shimizu Y, Ikada Y, Yamagishi H (2006). Tissue engineering of small intestinal tissue using collagen sponge scaffolds seeded with smooth muscle cells. Tissue Eng.

[148] Lee M, Wu BM, Stelzner M, Reichardt HM, Dunn JC (2008). Intestinal smooth muscle cell maintenance by basic fibroblast growth factor. Tissue Eng Part A.

[149] Watson CL, Mahe MM, Múnera J, Howell JC, Sundaram N, Poling HM (2014). An in vivo model of human small intestine using pluripotent stem cells. Nat Med.

[150] Zhu M, Li W, Dong X, Yuan X, Midgley AC, Chang H (2019). In vivo engineered extracellular matrix scaffolds with instructive niches for oriented tissue regeneration. Nat Commun.

[151] Stephenson M, Grayson W (2018). Recent advances in bioreactors for cell-based therapies. F1000Research.

[152] Mock U, Nickolay L, Philip B, Cheung GW, Zhan H, Johnston ICD (2016). Automated manufacturing of chimeric antigen receptor T cells for adoptive immunotherapy using CliniMACS prodigy. Cytotherapy.

[153] Nguyen B-NB, Ko H, Fisher JP (2016). Tunable osteogenic differentiation of hMPCs in tubular perfusion system bioreactor. Biotechnol Bioeng.

[154] Surrao DC, Boon K, Borys B, Sinha S, Kumar R, Biernaskie J (2016). Large-scale expansion of human skin-derived precursor cells (hSKPs) in stirred suspension bioreactors. Biotechnol Bioeng.

[155] Varley MC, Markaki AE, Brooks RA (2017). Effect of rotation on scaffold motion and cell growth in rotating bioreactors. Tissue Eng Part A.

[156] Grein TA, Leber J, Blumenstock M, Petry F, Weidner T, Salzig D (2016). Multiphase mixing characteristics in a microcarrier-based stirred tank bioreactor suitable for human mesenchymal stem cell expansion. Process Biochem.

[157] Martin I, Wendt D, Heberer M (2004). The role of bioreactors in tissue engineering. Trends Biotechnol.

[158] Ronaldson-Bouchard K, Vunjak-Novakovic G (2018). Organs-on-a-Chip: A fast track for engineered human tissues in drug development. Cell Stem Cell.

[159] Kim SS, Penkala R, Abrahimi P (2007). A perfusion bioreactor for intestinal tissue engineering. J Surg Res.

[160] Cei D, Costa J, Gori G, Frediani G, Domenici C, Carpi F (2016). A bioreactor with an electro-responsive elastomeric membrane for mimicking intestinal peristalsis. Bioinspir Biomim.

[161] Jeong SI, Kwon JH, Lim JI, Cho S-W, Jung Y, Sung WJ (2005). Mechano-active tissue engineering of vascular smooth muscle using pulsatile perfusion bioreactors and elastic PLCL scaffolds. Biomaterials.

[162] Harris LJ, Abdollahi H, Zhang P, McIlhenny S, Tulenko TN, DiMuzio PJ (2011). Differentiation of adult stem cells into smooth muscle for vascular tissue engineering. J Surg Res.

[163] Hayashi Y, Tsuji S, Tsujii M, Nishida T, Ishii S, Iijima H (2008). Topical implantation of mesenchymal stem cells has beneficial effects on healing of experimental colitis in rats. J Pharmacol Exp Ther.

[164] Qi D, Shi W, Black AR, Kuss MA, Pang X, He Y (2020). Repair and regeneration of small intestine: a review of current engineering approaches. Biomaterials.

[165] Forbes GM, Sturm MJ, Leong RW, Sparrow MP, Segarajasingam D, Cummins AG (2014). A phase 2 study of allogeneic mesenchymal stromal cells for luminal Crohn's disease refractory to biologic therapy. Clin Gastroenterol Hepatol.

[166] Duijvestein M, Vos ACW, Roelofs H, Wildenberg ME, Wendrich BB, Verspaget HW (2010). Autologous bone marrow-derived mesenchymal stromal cell treatment for refractory luminal Crohn's disease: results of a phase I study. Gut.

[167] Ciccocioppo R, Bernardo ME, Sgarella A, Maccario R, Avanzini MA, Ubezio C (2011). Autologous bone marrow-derived mesenchymal stromal cells in the treatment of fistulising Crohn's disease. Gut.

[168] Janis AD, Johnson CC, Ernst DM, Brightman AO (2012). Structural characteristics of small intestinal submucosa constructs dictate in vivo incorporation and Angiogenic response. J Biomater Appl.

[169] Mosala Nezhad Z, Poncelet A, De Kerchove L, Gianello P, Fervaille C, El Khoury G (2016). Small intestinal submucosa extracellular matrix (CorMatrix®) in cardiovascular surgery: a systematic review. Interact Cardiovasc Thorac Surg.

[170] Atala A, Bauer SB, Soker S, Yoo JJ, Retik AB (2006). Tissue-engineered autologous bladders for patients needing cystoplasty. Lancet.

[171] Joseph DB, Borer JG, De Filippo RE, Hodges SJ, McLorie GA (2014). Autologous cell seeded biodegradable scaffold for augmentation cystoplasty: phase II study in children and adolescents with spina bifida. J Urol.

[172] Meinhart JG, Deutsch M, Fischlein T, Howanietz N, Fröschl A, Zilla P (2001). Clinical autologous in vitro endothelialization of 153 infrainguinal ePTFE grafts. Ann Thorac Surg.

[173] Deutsch M, Meinhart J, Fischlein T, Preiss P, Zilla P (1999). Clinical autologous in vitro endothelialization of infrainguinal ePTFE grafts in 100 patients: a 9-year experience. Surgery.

[174] Elliott MJ, De Coppi P, Speggiorin S, Roebuck D, Butler CR, Samuel E (2012). Stem-cell-based, tissue engineered tracheal replacement in a child: a 2-year follow-up study. Lancet.

[175] Rouwkema J, Gibbs S, Lutolf MP, Martin I, Vunjak-Novakovic G, Malda J (2011). In vitro platforms for tissue engineering: implications for basic research and clinical translation. J Tissue Eng Regen Med.

[176] Lu L, Arbit HM, Herrick JL, Segovis SG, Maran A, Yaszemski MJ (2015). Tissue engineered constructs: perspectives on clinical translation. Ann Biomed Eng.

[177] Sloff M, Simaioforidis V, de Vries R, Oosterwijk E, Feitz W (2014). Tissue engineering of the bladder—reality or myth? A systematic review. J Urol.

[178] Colombo F, Sampogna G, Cocozza G, Guraya SY, Forgione A (2017). Regenerative medicine: clinical applications and future perspectives. J Microsc Ultrastruct.

